# Cigarette Smoking and Cardiovascular Risk in Young Women with Polycystic Ovary Syndrome

**Published:** 2013-12-22

**Authors:** Elena Morotti, Bruno Battaglia, Raffaella Fabbri, Roberto Paradisi, Stefano Venturoli, Cesare Battaglia

**Affiliations:** Department of Gynecology and Pathophysiology of Human Reproduction, Alma Mater Studiorum-University of Bologna, Bologna, Italy

**Keywords:** Smoke, PCOS, Ultrasound, Doppler

## Abstract

**Background:**

To verify if in lean polycystic ovary syndrome (PCOS) patients, the smok- ing habitude might increase the risk of cardiovascular (CV) disease.

**Materials and Methods:**

In this prospective observational study, eighty-one women were divided into the following three groups: group I with 27 non-smokers, group II with 26 light-smokers (1-10 cigarettes/day), and group III with 28 heavy smokers (>10 cigarettes/ day). They were submitted to fasting blood sampling; blood measurement of nitrites/ni- trates (NO_2_-/ NO_3_), biochemical and hormonal parameters; ovarian ultrasonographic (US) analysis; doppler evaluation of uterine and ophthalmic arteries; brachial artery flow-medi- ated vasodilatation; 24-hour ambulatory blood pressure monitoring; and oral glucose toler- ance test (OGTT).

**Results:**

Doppler analysis revealed higher uterine and ophthalmic arteries pulsatility in- dex (PI) and ophthalmic artery back pressure in group III compared with group I. The brachial artery diameter and PI, at baseline, was similar among all groups. After the re- active hyperemia, a more intense vasodilatation was observed in group I in comparison with group III. The 24-hour blood pressure demonstrated that, in group III patients, the 24-hour, day- and night-time diastolic blood pressure (DBP), was higher in comparison with non-smokers. The atherogenic index of plasma (AIP) was higher in heavy smokers than in non-smokers. The leukocytes and homocysteine (HCY) values were increased in group III. The NO_2_-/ NO_3_- plasma levels were reduced in heavy smokers in compari- son with non-smokers. The insulin, glucose and C-peptide plasma values were higher in group III than in other groups. In heavy smokers, the estimates of insulin sensitivity (ISI) and pancreatic β-cell function (HOMA-B) were higher compared to the other groups.

**Conclusion:**

Smoking habitude in lean PCOS patients may increase the soft markers of CV risk.

## Introduction

The polycystic ovary syndrome (PCOS) is one of the commonest endocrinopathy of premenopausal women (1). Insulin resistance is a well-recognized feature of PCOS and, in association with hypertension and dyslipidemia, may increase the risk of cardiovascular (CV) and cerebrovascular events (2-4). These risk factors are compounded by central obesity, which is a worsening and confounding factor present in the majority of women with PCOS (2). In the absence of adequate outcome studies, surrogate markers (i.e. increased carotid intima thickness, reduced brachial artery flow mediated vasodilatation, increased left ventricular mass, and increased homocysteine (HCY) and leukocyte levels, which provide a non-invasive assessment of arterial structure and function, have been evaluated to determine whether women with PCOS have evidence of subclinical CV disease as compared with controls. Despite the existence of no gold standard for the evaluation of the endothelial function, the measurement of the flow-mediated dilatation in the brachial artery is the most studied and promising method for clinical application. Endothelial dysfunction in peripheral arteries correlates with the presence of coronary artery endothelial dysfunction (5). Therefore, endothelial dysfunction in PCOS behaves as a marker for patients with preclinical vascular disease and may identify, at an early age, patients in whom therapeutic intervention could be beneficial. Pepene identified a platelet/endothelial cell adhesion molecule (PECAM)-1, with predictive value for endothelial dysfunction, increased in women with PCOS (6).

Cigarette smoking is a major health hazard. Cigarette smoke contains over 4,000 chemical constituents of which 60, at least, are toxic (7) and predispose to cancer, respiratory and CV diseases (8-10). Furthermore, smoking-associated changes in endocrine, metabolic and clinical features have been recently described in women with PCOS (11).

The individuation, in PCOS patients, of smoking specific CV effects may be of interest in counseling life-style modifications (smoking cessation, diet, physical exercise, blood pressure control, etc.).

To avoid age- and obesity-related bias, the aim of this study was to verify if in young lean PCOS patients, the smoking habitude might have an additive effect in worsening a risk of CV disease.

## Materials and Methods

Ninety-five young adult (18-35 years), lean (body mass index (BMI): 19-25 Kg/m2) Italian women with PCOS, referring to our clinic (Sant’Orsola- Malpighi Hospital, Bologna, Italy) for contraceptive necessities, were consecutively recruited into the present prospective observational study. The Rotterdam criteria (12) were used for the diagnosis of PCOS.

All women made no use of alcohol, psychoactive or recreational substances, did not take regular intense exercise, and did not receive hormonal therapy for at least 6 months prior to the study. In addition, women with known diabetes, renal or hepatic illness and with folic acid and vitamin B12 deficiencies were excluded from the study. The study protocol was in accordance with the Helsinki II declaration and was approved by the Hospital Research Review Committee. Women participated in the study after that an informed consent was obtained.

Twelve women were uninterested in completing the study. On the day of ultrasonographic (US) analysis, two subjects presented a persistent corpus luteum and were excluded from the study. Thus, 81 patients fulfilled the inclusion criteria and completed the study. On the basis of the smoking habit, the patients were divided into: group I with 27 nonsmokers, group II with 26 current (>2 years) light smokers (1-10 cigarettes/day; mean 4.3 ± 1.9/ day), and group III with 28 current heavy smokers (>10 cigarettes/day; mean 17.5 ± 5.6/day). The smoking duration was 6.5 ± 1.1 years and 8.8 ± 2.6 years, respectively, in group II and group III.

After the first screening evaluation, participants were assessed, in the early follicular phase (cycle days 3-5) with a detailed history and medical examination. Standing height and weight were measured and the mean BMI was calculated. A Ferriman- Gallwey score ≥8 indicated hirsutism.

Fasting blood samples were drawn for testing biochemical and hormonal parameters. Plasma concentrations of nitrites/nitrates (NO_2_-/ NO_3_) were also assayed (13). Patients were further submitted to utero-ovarian US analysis and to color doppler evaluation of uterine and ophthalmic arteries. In addition, US and color doppler analysis of brachial artery flow-mediated vasodilatation and 24- hour ambulatory blood pressure monitoring were performed. On the subsequent day, an oral glucose tolerance test (OGTT) was performed and blood was collected for the analysis of glucose, insulin and C-peptide. The lipid profile was studied.

US examination of the ovaries was performed with the use of a multi-frequency transvaginal transducer (RIC5-9H, Voluson 730 Expert Sonography System; GE Healthcare Ultrasound, Zipf, Austria). Ovarian volume, number and diameter of the follicles were recorded. Doppler flow measurements of the uterine arteries were performed transvaginally with a multi-frequency color doppler system (Voluson 730 Expert Sonography System Color Doppler, 5, 14). In addition, a color doppler analysis of ophthalmic arteries was performed using a multi-frequency linear array (SP10-16, Voluson 730 Expert Sonography System Color Doppler, 15, 16).

The pulsatility index (PI) was electronically calculated by the machine for the ovarian stromal, uterine and ophthalmic arteries. In view of the difficulty in interpreting the PI in low-impedance vascular beds such as the cerebral circulation (ophthalmic artery), downstream "back-pressure" was calculated using the model proposed by Gosling et al. (15).

The smoking habitude of the scanned patients was unknown. Ultrasound and color doppler analyses were performed by a single examiner (C.B.).

For the evaluation of the Brachial artery flow mediated vasodilatation, a high-resolution ultrasound transducer was placed over the brachial artery to measure its diameter before and after reactive hyperemia (17). Briefly, the right brachial artery was evaluated with continuous scanning held for 30 seconds using a multi-frequency linear array transducer (SP10-16, Voluson 730 Expert) over a longitudinal section 5-7 cm above the right elbow. A blood pressure cuff around the upper arm was then inflated to a pressure of 200 mmHg for 5 minutes. This caused ischemia and consequent dilatation of downstream resistance vessels. The subsequent cuff deflation induced a brief high-flow condition through the brachial artery (reactive hyperemia) due to the intense nitric oxide release from the endothelial cells. After this, scans were performed at 15 seconds, 60 seconds and 120 seconds. Flow mediated vasodilatation was also determined as the percentage change from baseline to 15 and subsequently to 120 seconds after sudden deflation to arm ischemia. A doppler analysis of brachial artery (Voluson 730 Expert Sonography System Color Doppler) was performed and the PIs registered (as absolute values and percentage variations) at baseline and just after US measurements of brachial artery.

Ambulatory blood pressure monitoring was performed using a portable lightweight device (Space lab 90121; Critikon, WA, USA) applied to the nondominant arm (18). Patients wore the device for 24 hours with measurements every 30 minutes during the day (08.00 a.m. to 10.00 p.m.) and hourly overnight (10.00 p.m. to 08.00 a.m.). The 24-hour blood pressure monitoring was considered statistically acceptable in presence of ≥75% successful measurements. Systolic blood pressure (SBP) and diastolic blood pressure (DBP) were calculated as 24- hour, day- and night-time variables. A blood pressure <130/80 mmHg for 24 hours was considered normal. The percentage of recordings exceeding these reference values was calculated. Two researchers (E.M., R.F.) separately analyzed the results.

### Assays

Peripheral blood flow was obtained from all patients between 8.00 a.m. and 11.00 a.m., after an overnight fast, on the same day that US and doppler examinations took place, and different hormonal and biochemical parameters were analyzed at the Sant’Orsola-Malpighi Hospital Central Laboratory, Bologna, Italy.

Plasma concentration of estradiol (E2), testosterone (T), androstenedione (A), 17-hydroxy-progesterone (17-OH-Pg) and sex hormone binding globulin (SHBG) were assayed (16, 19). The free androgen index (FAI) was calculated: FAI=T (nmol/l)/SHBG (nmol/l) ×100. Calculated free testosterone (cFT) was assessed using the formula available on a website of the International Society for the Study of the Aging Male (http//www.issam.ch/freetesto.htm). Hyperandrogenemia was defined as cFT ≥0.028 nmol/l (20). Results of hormonal values were converted to International System of Units (SI).

"The lipid profile (serum total cholesterol, high density lipoprotein (HDL) cholesterol and triglycerides) was studied. Low density lipoprotein (LDL) cholesterol was estimated as described by the Friedewald equation (21). The atherogenic index of plasma (AIP) was computed: AIP=log [triglycerides (mmol/l)/HDL-cholesterol (mmol/l)]" (22). Results of circulating lipids were converted to SI units.

Leukocyte count (n×10^3^) was determined within 2 hours after venipuncture.

An aliquot of peripheral blood was immediately centrifuged, and serum was stored at -70˚ until assays. Nitric oxide (NO) production was assessed by monitoring serum levels of stable oxidation products of NO metabolism (NO_2_-/ NO_3_). The NO_2_-/NO_3_- were assayed at Modena- Reggio Emilia University, Modena, Italy (13, 19). In addition, plasma HCY concentrations were determined with a method based on fluorescence polarisation immunoassay using Abbott (Imx, USA) analyzer (12).

On the subsequent day, after a further overnight fast, an OGTT (75 g Curvosio; Sclavo, Cinisello Balsamo, Italy) was performed, and blood was collected for the analysis of glucose, insulin and C-peptide at 15 minutes before and 30, 60, 90, and 120 minutes after the oral ingestion of glucose (23). Results, when necessary, were converted to SI units. The definition for normal fasting glucose, impaired fasting glucose, and diabetes were based on the established American Diabetes Association (ADA) criteria (24). Glucose tolerance was assessed by World Health Organization (WHO) criteria (23). Glucose, insulin and C-peptide determinations during the OGTT were used to calculate the respective areas under the curve (AUC_120_) at 120 minutes (3). The partial (0-90’ and 90’-120’) AUC_0-90_ and AUC_90-120_ were calculated. The homeostatic model assessment for insulin resistance (HOMA-IR), quantitative insulin sensitivity check index (QUICKI), insulin sensitivity index (ISI) and fasting glucose/insulin ratio were derived as estimates of insulin sensitivity. In addition to the fasting C-peptide and insulin levels, the insulinogenic index and the homeostatic model assessment for percent pancreatic β-cell function (HOMA-B) were derived as indices of pancreatic β-cell function. For the same purposes, β-cell secretion of insulin was estimated by the following indices: predicted indexes of 1^st^ and 2^nd^ phase of insulin secretion (1^st^ and 2^nd^ PHIS). The fasting C-peptide/ insulin molar ratio was considered an index of hepatic insulin clearance (3).

### Statistical analysis

Statistical analysis (SPSS 11.5 software; SPSS inc., Chicago IL, USA) was performed using the one-way ANOVA with Bonferroni’s post-hoc correction. The relationship between the parameters was analyzed using the Spearman’s nonparametric correlation. A p value ≤0.05 was considered as statistically significant. Data are presented as mean ± SD, unless otherwise indicated. The statistical analysis was performed by a single researcher (B.B.).

## Results

All 81 women completed the study. The three groups of studied patients, on the basis of inclusion criteria, did not differ in age and BMI. The age at menarche was similar in the observed groups of patients (Table 1). The Ferriman-Gallwey score was as indicated in table 1.

**Table 1:** Physical, clinical, and hormonal profile in non smoking (Group I) versus light (Group II) and heavy smoking (Group III) PCOS patients


	Group I (n=27)	Group II (n=26)	Group III (n=28)	Significance		
							I vs II	I vs III	II vs III

**Age (yrs)**	26.1±5.2	25.5±3.2	26.5±4.2			
**BMI (Kg/m^2^)**	22.4±2.8	22.9±1.9	23.3±4.0			
**Ferriman-Gallwey score**	12.7±5.1	12.9±5.9	13.8±4.7			
**Age at menarche (yrs)**	11.7±1.1	11.4±1.4	12.0±2.3			
**Estradiol (pmol/l)**	169±91	146±71	199±84			
**Androstenedione (nmol/l)**	12.0±4.5	12.7±4.6	15.5±4.9		0.039	
**17-OH-Progesterone (nmol/l)**	4.6±2.6	4.4±2.1	5.2±3.0			
**Testosterone (nmol/l)**	1.8±1.0	2.0±1.3	2.1±1.7			
**SHBG (nmol/l)**	50.0±18.5	43.4±17.4	40.9±18.3			
**FAI (%)**	5.6±4.2	6.7±4.2	7.1±5.6		0.0312	
**cFT (nmol/l)**	0.029±0.002	0.034±0.002	0.036±0.003		0.027	


The plasma levels of E2, T, A, and 17-OH-Pg are reported in table 1. The SHBG values, the FAI and the cFT resulted as reported in table 1.

At the US evaluation, the mean ovarian volume and the mean number of small subcapsular follicles were not significantly different among the three groups (Table 2). At doppler analysis, a significantly higher mean uterine PI was found in group III (3.16 ± 0.79) compared with group I (2.37 ± 0.64; p=0.003). No significant differences were observed between light smokers (group II) and non-smokers (group I). The mean PI of ophthalmic arteries was higher in light and heavy smokers than in non-smokers. Also, the ophthalmic artery back-pressure was significantly higher in group II (62 ± 4 mmHg; p=0.007) and group III (65 ± 5 mmHg; p<0.001) than in group I (51 ± 9 mmHg, Table 2).

**Table 2:** Ultrasonographic, Doppler measures and 24 h blood pressure monitoring in non smoking (Group I) versus light (Group II) and heavy smoking (Group III) PCOS patients


	Group I (n=27)	Group II (n=26)	Group III (n=28)	Significance		
							I vs II	I vs III	II vs III

**Ovarian Volume (ml)**	12.3±2.0	13.0±2.1	13.3±1.7			
**Follicles (n)**	15.8±5.8	13.8±2.7	13.8±4.0			
						
**Uterine Artery (PI)**	2.37±0.64	2.64±0.95	3.16±0.79		0.003	
**Ophthalmic Artery (PI)**	1.72±0.45	2.09±0.56	2.10±0.48	0.034	0.027	
**Ophthalmic Back Pressure (mm/Hg)**	51±9	62±4	65±5	0.007	<0.001	
**24 h DBP (mm/Hg)**	68±5	69±5	73±7		0.027	
**08-22 h SBP (mm/Hg)**	114±5	115±9	112±9			
**08-22 h DBP (mm/Hg)**	70±6	72±6	76±7		0.003	
**22-08 SBP (mm/Hg)**	103±5	105±9	106±9			
**22-08 DBP (mm/Hg)**	59±5	62±7	65±7		0.050	
**24 h % SBP > 130 mmHg**	9±4	9±7	11±3			
**24 h % DBP > 80 mm/Hg**	12±8	17±5	17±4			


**Fig 1:**
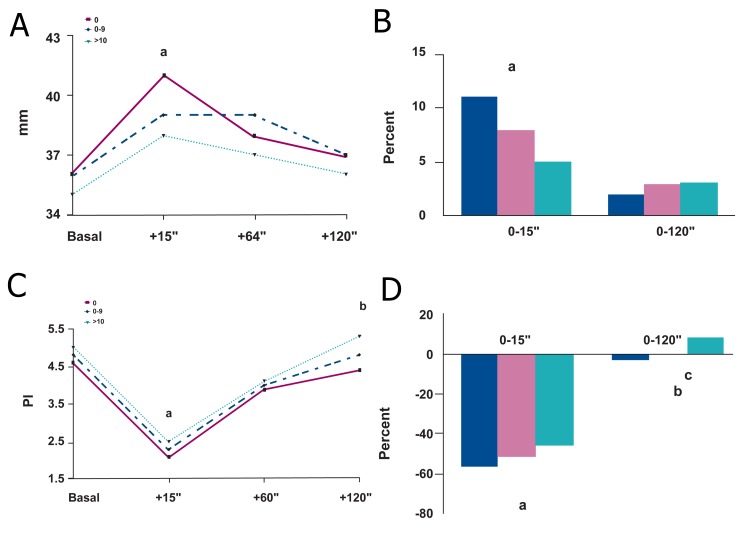
Brachial artery ultrasonographic analysis (Left). A. (upper panel). Brachial artery diameter was similar, at baseline, in all patients. After reactive hyperemia (15 seconds), a more intense vasodilatation (a; p=0.048) was observed in non-smokers. Thereafter, and since the end of the study (120 seconds), no further differences were evidenced. B. (lower panel). The percentage changes 15 seconds after reactive hyperemia was lower in heavy smoking than in non-smoking patients (b; p=0.012). At 120 seconds, the value was similar in all groups. Brachial artery doppler analysis (Right). C. (upper panel). At baseline, the pulsatility index (PI) was similar in all patients. After reactive hyperemia (15 seconds), a more intense vasodilatation (a; p=0.033) was observed in non-smokers in comparison with heavy smokers. The persistence of reactive hyperemia was more evident in non-smokers than in heavy smokers (b; p=0.011). D. (lower panel). The percentage changes 15 seconds after reactive hyperemia was lower in heavy smoking than in non-smoking patients (a; p=0.049). At 120 seconds, reactive hyperemia was significantly reduced in heavy smokers than in light smokers (c; p=0.039) and in non-smokers (b; p=0.023).

The brachial artery diameter, at baseline, was similar in all the patients (Fig 1). After the reactive hyperemia, a more intense vasodilatation was observed in group I in comparison with group III. The persistence of the effect was not significantly different among the groups. The percentage change at 15-second was significantly lower in group III than in group I. At 120-second, the reactive hyperemia was similar in all patients. At baseline, the PI of the brachial artery was slightly, but not significantly, higher (5.0 ± 1.5) in group III than in group II (4.8 ± 1.4) and group I (4.6 ± 1.6). The doppler variations at level of the brachial artery were more evident and persistent in non-smokers than in heavy smokers. The percentage change at 15-second was significantly higher in group I than group III. At 120-second, the reactive hyperemia was still kept only in non-smokers and light smokers (Fig 1).

The 24 hour blood pressure monitoring, among the groups, showed no significant differences in the 24-hour, day- and night-time SBP values. However, in group III patients, the 24 hour, day- and night-time DBP, was, although in the normal range, significantly higher in comparison with non-smokers. The percentage of recordings, exceeding a blood pressure of 130/80 mmHg for the 24 hour, was similar among the groups (Table 2).

**Fig 2:**
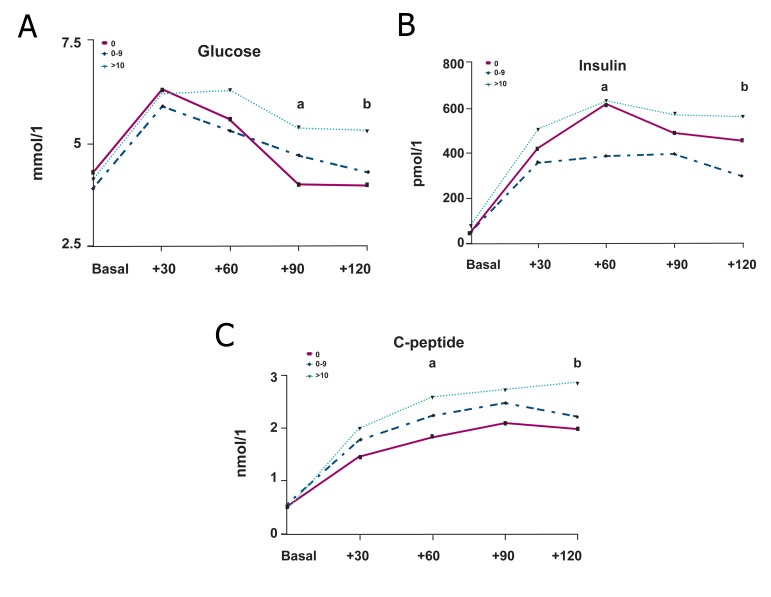
Oral glucose tolerance test (OGTT). A. (upper left panel). Glucose during OGTT. The circulating glucose levels, at 90 and 120 minutes after oral glucose load, were significantly higher in heavy smokers than nonsmokers (a; p=0.019; b; p=0.001). B. (upper right panel). Insulin during OGTT. The plasma insulin levels, at 60 and 120 minutes, were significantly higher in heavy smokers than non-smokers (a; p=0.032; b: p=0.017). C. (lower panel). C-peptide during OGTT. The circulating C-peptide levels, at 60 and 120 minutes, were significantly higher in heavy smokers than non-smokers (a; p=0.047; b; p=0.035).

Total cholesterol, HDL and LDL cholesterol and triglycerides were not significantly different among the three groups. However, the AIP was significantly higher in heavy smokers than in non-smokers (Table 3). The leukocytes were slightly, but significantly, increased in group III (7,400 ± 1,800) in comparison with group II (6,200 ± 1, 200; p=0.049) and group I (6,100 ± 1,700; p=0.035). The HCY was slightly higher in group III (11.2 ± 2.7 μmol/l) than in group I (9.3 ± 2.8 μmol/l; p=0.031). The NO_2_-/ NO_3_- plasma levels were significantly reduced in heavy smokers in comparison with nonsmokers (Table 3). Fasting values of glucose, insulin, and C-peptide are reported in table 3. The insulin plasma resulted in higher values in heavy smokers than in other groups. The glucose, insulin, and C-peptide plasma values after the OGTT are reported in fig 2. The area under the curve (AUC_120_) for glucose, insulin and C-peptide are shown in table 3: the group III patients presented the worst results. Furthermore, these patients presented higher values also in the later partial (AUC_90-120_) glucose, insulin and C-peptide areas under the curve. The different estimates (HOMAIR, QUICKI, ISI, and glucose/insulin ratio) of insulin sensitivity and indices of pancreatic β-cell function (HOMA-B, insulinogenic index, 1^st^ and 2nd PHIS) are reported in table 3. In the same table (Table 3), the values of the fasting C-peptide/insulin molar ratio (as an index of hepatic insulin clearance) are shown.

The relation between smoking and studied parameters is reported in table 4.

**Table 3:** Metabolic Profile in non smoking (Group I) versus light (Group II) and heavy smoking (Group III) PCOS patients


				Significance		
Group I (n=27)	Group II (n=26)	Group III (n=28)	I vs II	I vs III	II vs III

**Total Cholesterol (mmol/l)**	175±45	160±16	165±33			
**HDL Cholesterol (mmol/l)**	61±13	61±12	58±10			
**LDL Cholesterol (mmol/l)**	96±39	87±13	89±26			
**Triglycerides (mmol/l)**	91±71	73±29	100±54			
**AIP (nmol/l)**	-0.36±0.05	-0.27±0.38	-0.17±0.05		0.028	
**Leucocytes (n x 1,000)**	6.3±1.7	6.2±1.2	7.4±1.8		0.035	0.049
**Homocysteine (μmol/l)**	9.3±2.8	9.9±2.7	11.2±2.7		0.031	
**NO_2_-/ NO_3_- (μmol/l)**	28.4±6.9	23.6±6.7	21.4±6.6		0.049	
**Insulin AUC_0-90_ (pmol/l)**	42170±27050	66210±37500	63333±32910			
**Insulin AUC_90-120_ (pmol/l)**	15160±11080	20830±15750	26701±15750		0.002	
**C-peptide AUC_120_ (nmol/l)**	234±81	282±93	326±94		0.009	
**C-peptide AUC_0-90_ (nmol/l)**	141±51	177±71	185±77			
**C-peptide AUC_90-120_ (nmol/l)**	63±25	73±29	89±24		0.011	
**HOMA-IR **	1.2±1.7	2.0±1.8	2.1±1.4		0.037	
**QUICKI**	0.386±0.043	0.381±0.052	0.373±0.064			
**ISI**	0.11±0.03	0.10±0.02	0.09±0.03			
**Fasting Glucose/Insulin Ratio**	12.3±0.7	10.4±0.4	7.2±0.4		<0.001	
**HOMA-B**	185±61	199±76	255±50		0.022	
**Insulinogenic Index**	0.07±0.04	0.08±0.04	0.13±0.07		0.006	0.014
**Ist PHIS**	1586±447	1278±487	1079±317		0.047	
**2^n^^d^ PHIS**	464±75	380±80	324±97		0.043	
**Fasting C-peptide/Insulin Ratio**	11.6±2.6	11.8±2.6	8.6±3.0		0.033	0.027


**Table 4:** Relationship between cigarettes and different measures, analyzed using Spearman's Nonparametric Correlation


	ρ	p

**Androstenedione**	0.209	0.050
**FAI**	0.373	0.001
**AIP**	0.331	0.001
**NO_2_-/ NO_3_- **	-0.466	<0.001
**Lecocytes**	0.363	0.001
**Uterine Artery PI**	0.284	0.031
**Ophthalmic Artery PI**	0.396	0.001
**Ophthalmic Artery Back Pressure**	0.396	0.001
**Brachial Artery PI**	0.214	0.047
**Fasting Insulin**	0.236	0.042
**Insulin AUC_120_**	0.276	0.030
**Insulinogenic Index**	0.264	0.029


## Discussion

There are mounting suggestions that PCOS women may have an increased risk of cardio- and cerebro-vascular pathologies in comparison with normal cycling women of similar age and BMI (3, 25). In western countries, more than 30% of reproductive age women smoke cigarettes (26). Epidemiological studies strongly support the assertion that cigarette smoking increases the incidence of myocardial infarction, fatal coronary artery disease, peripheral vascular disease, and stroke (8).

In the present study, the heavy smokers presented significantly higher A, FAI and cFT circulating values in comparison with non-smokers. Furthermore, cigarette smoking was directly correlated with A and FAI. Cigarettes may, in a dosedependent manner, interfere with steroid hormone release, binding, transport, storage, metabolism and clearance, resulting in changes in circulating hormone concentrations. Specifically, nicotine and anabasine were found to inhibit granulosa cell aromatase with increasing A circulating levels (27). Furthermore, the association between nicotine and calculated cFT may be related to the increased activity of cyotochrome p450, which is involved in both the metabolic pathway of T and nicotine (28).

All patients were studied in a hypoestrogenic state (early follicular phase); however, the highest uterine vascular resistances resulted in heavy smoking women. We speculated that the worst uterine vascularization might be due to the more elevated androgen circulating levels. In fact, androgens have direct vasoconstrictive effects on vascular tissue, mediated by specific receptors present in the main arterial blood vessels walls (29). Among the three groups of patients, we also analyzed the hemodynamic properties of ophthalmic arteries (16, 30). The ophthalmic artery is a small vessel arising from the internal carotid artery. Cerebral vessels are morphologically and physiologically similar to the arteries of the eye. Thus, knowledge of vascular changes in the ophthalmic arteries may be useful in assessing changes in global cerebral perfusion (31, 32). Our study showed higher ophthalmic artery PI values and ophthalmic artery back-pressure (the sum of arteriolar vasomotor tone and intracranial pressure, 15) in smokers than in non-smokers. Our findings on ocular/cerebral vascularization were correlated with smoking attitude. We speculated that the increased arterial stiffness, the atherogenic lipid profile and the lower circulating NO_2_-/ NO_3_- values may be responsible of the increased ophthalmic PIs and back pressure in PCOS smoking women. In addition, smoking may aggravate insulin resistance (11), and this is widely considered related to type II diabetes, in which regional cerebral blood flow is reduced (33). These data may in part explain the increased risk of cerebro-vascular pathologies in smoking patients.

The vascular endothelium is a complex organ. Abnormalities in endothelial function have been associated with several CV risk factors and may portend clinically significant vascular diseases. In addition, endothelial dysfunction precedes overt vascular disease by years. Despite no gold standard exists for the evaluation of endothelial function, the measurement of the flow-mediated dilatation in the brachial artery is the most studied and promising method for clinical application (34). In our study, we observed a more intense post-ischemic vasodilatation in non-smoker in comparison with heavy smokers. Similar results were observed in performing the doppler analysis of brachial artery PIs. A positive relation was reported between smoking and brachial artery reactivity. The NO_2_-/ NO_3_ plasma levels resulted in a significant reduction in heavy smoking patients in respect to the non-smokers. A significant inverse relation was observed between smoking and NO_2_-/NO_3_ circulating plasma levels (ρ=-0.446; p<0.001). Thus, we derived that smoking negatively influence the brachial artery flow mediated vasodilatation and, furthermore, that smoking is associated with a reduction of nitric oxide release/production or to an increased nitric oxide degradation and to an impaired endothelium-dependent vasodilatation (9). Therefore, endothelial dysfunction in PCOS smokers behaves as a marker for patients with preclinical vascular disease and may identify, at an early age, patients in whom therapeutic intervention could be beneficial.

It is controversial if smoking "per se" is associated with hypertension. Using the 24-hour ambulatory monitoring, we demonstrated, in heavy smokers, an increased 24 hour, daytime and nighttime DBP. This rather labile control of the DBP might indicate a very precocious pre-hypertensive state. With advancing age, apart from some specific factors due to genetics, inactivity, obesity, stress, and salt loading, it is plausible that blood pressure may increase at an accelerated rate in PCOS smokers given the stimulatory effects of hyperinsulinemia on the sympathetic nervous system and vascular smooth muscle, and the changes noted in the endothelial function.

In the present study, we also found an increased leukocyte count in heavy smokers in comparison with light smokers and non-smokers. Inflammation has been recognized to have a pivotal role in both initiation and progression of the atherosclerotic processes. Indeed, increased white blood cells count is directly associated with an increased incidence of myocardial infarction and ischemic stroke. Hyper-HCY levels are considered too as an independent risk factor for CV diseases. In our study, more elevated serum HCY levels were observed in heavy smokers. As a result of these findings, we can suggest that PCOS and smoking may concur in inducing hyper-HCY and in increasing the risk of CV diseases.

The AIP has been proposed as a marker of the atherogenic potential of the plasma. In our study, the AIP was significantly higher in heavy smokers than in other groups. AIP resulted in a positive correlation with smoking, underlining the deep interdependence between smoking and the lipid profile.

PCOS is considered to be a metabolic disorder. Legro et al. (35) have suggested that women with PCOS are at significantly increased risk for impaired glucose intolerance and diabetes type 2 at all weight levels and at a young age, while they have a greater than 2-fold relative risk for myocardial infarction and micro- and macro-vascular disease. Gharakhani et al. (36) reported that the administration of metformin reduce the CV risk in PCOS women. Recently, Cupisti et al. (11) demonstrated that, in PCOS women, the smoking habitude is associated with increased fasting insulin levels and aggravated insulin resistance. In our study, we observed that among the estimates of insulin sensitivity, the HOMA-IR was significantly higher, whereas the fasting glucose/insulin ratio was lower in heavy smokers than in other groups. In addition to the fasting insulin levels, the insulinogenic index and the HOMA-B (all indices of pancreatic β-cell function) resulted in significantly higher value for PCOS smoking patients than weight matched non-smokers. Furthermore, the fasting C-peptide-to-insulin molar ratio (a useful surrogate of hepatic insulin clearance) was lower in heavy smokers than in other groups. During OGTT, plasma insulin was significantly higher in smokers than in non-smokers. The insulin total AUC_120_ was significantly more elevated in PCOS heavy smoking patients than in non-smoking women. A similar relationship was observed for the late partial insulin AUC_90-120_. The glucose and C-peptide expressed similar tendencies. In addition, the fasting insulin, the insulin total AUC_120_, and ISI were significantly correlated with cigarette smoking. We speculated that in PCOS patients, the cigarette smoking might induce a moderate-to-severe muscle insulin resistance and also a concomitant liver insulin resistance. This, associated with the increased pancreatic β-cell function and the reduced hepatic insulin clearance we observed, may explain hyperinsulinemia in those PCOS patients who smoke and their higher risk for progression to type-2 diabetes and CV diseases.

Smoking is "per se" an important risk factor for CV diseases. Wang et al. associated pack-years of smoking with the severity of angiographically determined atherosclerosis (37). Furthermore, thoracic aortic atherosclerotic lesions have been increased in smokers vs. nonsmokers. In addition, it has also been reported that cigarette smoking is associated with a significant increase of carotid intima-media thickness and carotid stiffness (9). On the other hand, PCOS is also "per se" a significant risk factor for cardio-vascular disease (3). As far as our knowledge, there are no previous studies considering both PCOS and smoking, the simultaneous presence of both these factors, which increases significantly the cardio-vascular risk in comparison with both healthy smokers and non-smokers PCOS patients.

## Conclusion

On the basis of the data presented, we postulate that, in PCOS patients, the smoking habitude may worsen the vascular reactivity, the carbohydrate and lipids metabolism and, consequently, the CV risk. Therefore, it is very important to lead PCOS patients to stop smoking in order to reduce the CV risk.

## References

[1] Asgharnia M, Mirblook F, Soltani MA (2011). The prevalence of polycystic ovary syndrome (PCOS) in high school students in Rasht in 2009 according to NIH Criteria. Int J Fertil Steril.

[2] Meyer C, McGrath BP, Teede HJ (2005). Overweight women with polycystic ovary syndrome have evidence of subclinical cardiovascular disease. J Clin Endocrinol Metab.

[3] Battaglia C, Mancini F, Cianciosi A, Busacchi P, Facchinetti F (2008). Vascular risk in young women with polycystic ovary and polycystic ovary syndrome. Obstet Gynecol.

[4] Mani H, Levy MJ, Davies MJ, Morris DH, Gray LJ, Bankart J (2013). Diabetes and cardiovascular events in women with polycystic ovary syndrome; a 20-years retrospective cohort study. Clin Endocrinol.

[5] Battaglia C, Mancini F, Cianciosi A, Busacchi P, Persico N, Paradisi R (2009). Cardiovascular risk in normal weight, eumenorrheic, nonhirsute daughters of patients with polycystic ovary syndrome: a pilot study. Fertil Steril.

[6] Pepene CE (2012). Soluble platelet/endothelial cell adhesion molecule (sPECAM)-1 is increased in polycystic ovary syndrome and related to endothelial dysfunction. Gynecol Endocrinol.

[7] Shiverik KT, Salafia C (1999). Cigarette smoking and pregnancy I: ovarian, uterine and placental effects. Placenta.

[8] Ambrose JA, Barua RS (2004). The pathophysiology of cigarette smoking and cardiovascular disease: an update. J Am Coll Cardiol.

[9] Battaglia C, Battaglia B, Mancini F, Persico N, Nappi RE, Paradisi R (2011). Cigarette smoking decreases the genital vascularization in young healthy, eumenorrheic women. J Sex Med.

[10] Hjellvik V, Selmer R, Gjessing HK, Tverdal A, Vollset SE (2013). Body mass index, smoking, and risk of death between 40 and 70 years of age in a norwegian cohort of 32,727 women and 33,475 men. Eur J Epidemiol.

[11] Cupisti S, Haberle L, Dittrich R, Oppelt PG, Reissmann C, Kronawitter D (2010). Smoking is associated with increased free testosterone and fasting insulin levels in women with polycystic ovary sysndrome, resulting in aggravated insulin resistance. Fertil Steril.

[12] The Rotterdam ESHRE/ASRM-sponsored PCOS consensus workshop group (2004). Revised 2003 consensus on diagnostic criteria and long-term health risks related to polycystic ovary syndrome. Fertil Steril.

[13] Facchinetti F, De Martis S, Neri I, Caputo A, Volpe A (1997). Effects of transdermal glyceryltrinitrate on 24-h blood pressure changes in patients with gestational hypertension. Prenat Neonat Med.

[14] Battaglia C, Artini PG, D’Ambrogio G, Genazzani AD, Genazzani AR (1995). The role of color doppler imaging in the diagnosis of polycystic ovary syndrome. Am J Obstet Gynecol.

[15] Gosling RG, Lo PT, Taylor MG (1991). Interptretation of pulsatility index in feeder arteries to law-impedance vascular beds. Ultrasound Obstet Gynecol.

[16] Battaglia C, Mancini F, Regnani G, Persico N, Volpe A, De Aloysio D (2004). Hormone therapy and ophtalmic artery blood flow changes in women with primary open-angle glaucoma. Menopause.

[17] Corretti MC, Anderson TJ, Benjamin EJ, Celermajer D, Charbonneau F, Creager MA (2002). Guidelines for the ultrasound assessment of endothelial-dependent flowmediated vasodilation of the brachial artery: a report of the International Brachial Artery Reactivity Task Force. J Am Coll Cardiol.

[18] Staessen JA, Fagard R, Thijs L, Amery A (1995). A consensus view on the technique of ambulatory blood pressure monitoring.The fourth international consensus conference on 24-hour ambulatory blood pressure monitoring. Hypertension.

[19] Battaglia C, Regnani G, Marsella T, Facchinetti F, Volpe A, Venturoli S (2002). Adjuvant L-arginine treatment in controlled ovarian hyperstimulation: a double-blind, randomized study. Hum Reprod.

[20] Vermeulen A, Verdonk L, Kaufman JM (1999). A critical evaluation of simple method for the estimation of free testosterone in serum. J Clin Endocrinol Metab.

[21] Friedewald WT, Levy RI, Fredrickson DS (1972). Estimation of the concentration of low-density lipoprotein cholesterol in plasma, without use of the preparative ultracentrifuge. Clin Chem.

[22] Lambrinoudaki I, Christodoulakos G, Rizos D, Economou E, Argeitis J, Vlachou S (2006). Endogenous sex hormones and risk factors for atherosclerosis in healthy Greek postmenopausal women. Eur J Endocrinol.

[23] Modan M, Harris MI, Halkin H (1989). Evaluation of WHO and NDDG criteria for impaired glucose tolerance.Results from two national samples. Diabetes.

[24] American diabetes association (1997). clinical practice recommendations. Diabetes Care.

[25] Dahlgren E, Janson PO, Johansson S, Lapidus L, Oden A (1992). Polycystic ovary syndrome and risk for myocardial infarction.Evaluated from a risk factor model based on a prospective population study of women. Acta Obstet Gynecol Scand.

[26] Practice Committee of the American Society for Reproductive Medicine (2008). Smoking and infertility. Fertil Steril.

[27] Soldin OP, Makambi KH, Soldin SJ, O’Mara DM (2011). Steroid hormone levels associated with passive and active smoking. Steroids.

[28] Ray R, Tyndale RF, Lerman C (2009). Nicotine dependence pharmacogenetics: role of genetic variation in nicotine-metabolizing enzymes. J Neurogenet.

[29] Chattopadhyay K, Chattopadhyay BD (2008). Effect of nicotine on lipid profile, peroxidation and antioxidant enzymes in female rats with restricted dietary protein. Indian J Med Res.

[30] Battaglia C, Mancini F, Fabbri R, Persico N, Busacchi P, Facchinetti F (2010). Polycystic ovary syndrome and cardiovascular risk in young patients treated with dospirenoneethinylestradiol or contraceptive vaginal ring.A prospective, randomized, pilot study. Fertil Steril.

[31] Powers WJ, Press GA, Grubb RL Jr, Gado M, Raichle ME (1987). The effect of hemodynamically significant carotid artery disease on the hemodynamic status of the cerebral circulation. Ann Intern Med.

[32] Ikram MK, Witteman JCM, Vingerling JR, Breteler MM, Hofman A, De Jong PT (2006). Retinal vessel diameters and risk of hypertension: the Rotterdam study. Hypertension.

[33] Wakisaka M, Nagamachi S, Inoue K, Morotomi Y, Nunoi K, Fujishima M (1990). Reduced regional cerebral blood flow in aged noninsulin-dependent diabetic patients with no history of cerebrovascular disease: evaluation by N-isopropyl- 123I-p-iodoamphetamine with single-photon emission computed tomography. J Diabet Complications.

[34] Faulx MD, Wright AT, Hoit BD (2003). Detection of endothelial dysfunction with brachial artery ultrasound scanning. Am Heart J.

[35] Legro RS, Gnatuk CL, Kunselman AR, Dunaif A (2005). Changes in glucose tolerance over time in women with polycystic ovary syndrome: a controlled study. J Clin Endocrinol Metab.

[36] Gharakhani M, Neghab N, Farimani M (2011). Is reducing ovarian volume in polycystic ovarian syndrome patients after administration of metformin associated with improving cardiovascular risk factors. Int J Fertil Steril.

[37] Wang XL, Tam C, McCredie RM, Wilcken DE (1994). Determinants of severity of coronary artery disease in Australian men and women. Circulation.

